# Physics-based data augmentation to enhance deep learning performance in tropical cyclone and storm surge modelling

**DOI:** 10.1038/s41598-026-58153-2

**Published:** 2026-06-22

**Authors:** Takumu Iwamoto, Tomohiro Takagawa

**Affiliations:** Tsunami and Storm Surge Group, Port and Airport Research Institute, Yokosuka, Japan

**Keywords:** Storm surges, Tropical cyclones, Machine learning, Fine-tuning, Data augmentation, Climate sciences, Natural hazards

## Abstract

Accurate modelling of tropical cyclones (TCs) is essential for reliable storm surge simulations, as TCs provide the primary external forcing. While deep learning models have demonstrated effectiveness in TC modelling, a challenge remains regarding the limited diversity of TCs in reanalysis data used for training—specifically, the scarcity of extreme TC events. This study proposes a physics-based data augmentation method that utilizes a numerical weather prediction (NWP) model to physically generate TCs lying beyond the range of reanalysis data. Subsequently, focusing on small-sized yet intense TCs as extreme cases, a model—originally pre-trained solely on reanalysis data—was fine-tuned using this augmented dataset to convert a parametric TC model (PM) field into an NWP-like field. Validation using test data mimicking extreme TCs and a storm surge hindcast of TC Faxai (a compact, intense TC that struck Tokyo Bay in 2019) revealed that the PM failed to simulate storm surges where topographic effects on the wind field are significant, and the pre-trained model underestimated wind speeds and storm surges. In contrast, the fine-tuned model successfully captured the spatiotemporal features of the extreme TCs and the peak storm surges upon TC landfall, achieving the lowest RMSE for storm surges across all TCs and tide gauges. These results suggest that physics-based data augmentation can effectively extend the applicability of deep learning models for TC and storm surge modelling to extreme events.

**Supplementary Information:**

The online version contains supplementary material available at https://doi.org/10.1038/s41598-026-58153-2.

## Introduction

In the numerical simulation of storm surges, wind and pressure fields generated by tropical cyclones (TCs) are applied as external forcing. Typically, either a parametric model (PM) or a physics-based numerical weather prediction (NWP) model is used^1^. PMs can simulate TC fields at a low computational cost using a limited set of key parameters of TCs (i.e., location, central pressure, etc.); however, they do not account for effects such as wind field deformation due to landfall. In contrast, NWP models offer the advantage of physically simulating TC behavior, although they are computationally expensive and do not always reproduce TC intensity with high accuracy^2^. Consequently, methods for correcting TC intensity have been proposed and applied to storm surge simulations in various coastal areas^3–6^. These studies often employ blending techniques^7^ that utilize NWP for the TC’s outer regions and PM for the center’s vicinity; however, this approach is not always effective in improving the accuracy of storm surges near the TC’s center, as PMs ignore topographic effects on the wind fields^8^.

In this context, a recent study^9^ reported using deep learning to correct PMs, allowing them to approximate NWP meteorological fields for application in storm surge simulations. AI-based TC modelling is advancing rapidly; for instance, forecast models such as Pangu-Weather^10^ and GraphCast^11^ have occasionally outperformed NWPs in TC track prediction accuracy^12^, and the European Centre for Medium-Range Weather Forecasts (ECMWF) operationalized global-scale AI weather forecasting in 2024^13^. In addition, generative AI is being utilized for the downscaling of meteorological fields, including complex TC structures^14–16^. The computational efficiency of AI is another powerful advantage, enabling large-scale ensemble TC track forecasting that incorporates physics-based perturbations^17^. These advancements underscore the utility of AI in TC modelling.

**Fig. 1:**
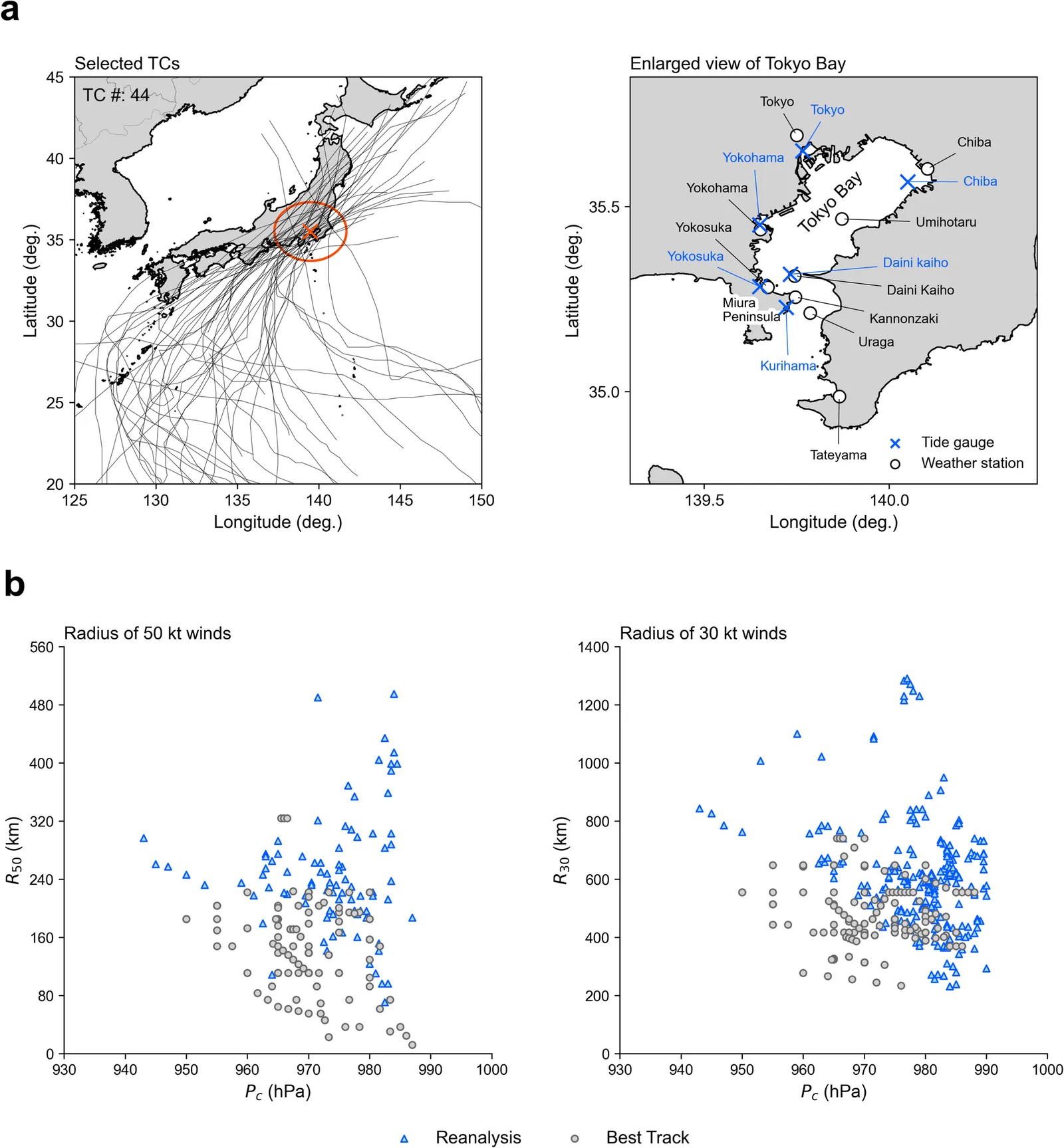
(a) TCs approaching the vicinity of Tokyo Bay. The left panel shows TC tracks (gray solid lines) that passed within a 200 km radius (circle) of the reference point (139.5°E, 35.5°N) near Tokyo Bay between 1958 and 2012, the period covered by DSJRA-55. The right panel shows the locations of observation points around Tokyo Bay. (b) Relationship between central pressure and the radii of 50 kt and 30 kt winds. DSJRA-55 is labeled as “Reanalysis” and IBTrACS^19^ as “Best Track.” Statistics show the selected TCs located between 34 N and 36 N, using identical timesteps for both datasets.

However, when modelling TCs using AI, the limited diversity of TCs included in the training data can be a significant concern. For instance, in the DSJRA-55 dataset^18^(used in the prior research^9^ for its ability to resolve TC intensity and track), discrepancies exist between DSJRA-55 and the best track^19^ regarding the radii of 50 kt and 30 kt winds for TCs approaching the Tokyo Bay area (Fig. 1(a)). This discrepancy is evident for TCs with small 50 kt and 30 kt wind radii (Fig. 1(b)). If such data is used for training, the resulting model may not accurately reproduce extreme TCs located beyond the range of the training data. Notably, since NWPs often exhibit systematic underestimation bias in reproducing TC intensity^2^, especially at the stage of rapid intensification^20^, the issue regarding the limited diversity of TCs can be common^21^ in other reanalysis datasets such as ERA-5^22^.

To address the issue, data augmentation offers a potential solution. In computer vision, standard augmentation techniques rely on image transformations such as flipping, rotation, and translation^23^. While these techniques have proven effective for estimating TC intensity^24^, they merely augment existing data patterns; consequently, the issue arising from the limited diversity of TCs remains unresolved. Therefore, we propose a physics-based data augmentation method to generate TCs absent from existing reanalysis data. Specifically, spin-up simulations of an axisymmetric TC model^25^ were performed using the Weather Research and Forecasting (WRF) model^26^, with the results being embedded into the computational domain of the target reanalysis data. Advection simulations of embedded TCs^27^ were then conducted to create an augmented dataset. The novelty of this study lies in its focus on generating intense TCs with narrow wind fields—events rarely captured in reanalysis data. Subsequently, following the prior study^9^, the deep learning model Pix2Pix^28^ was pre-trained on DSJRA-55 and fine-tuned using the augmented data. Finally, the impact of fine-tuning on the accuracy of simulating TCs and storm surges was evaluated under two different scenarios: a hypothetical scenario using the augmented test dataset and a hindcast of TC Faxai (2019) striking Tokyo Bay. Tokyo Bay is a semi-enclosed shallow water area facing the Pacific Ocean (Fig. 1(a)), where significant storm surges generated by TCs frequently occur^29^. TC Faxai is documented as an extreme case of a compact, intense TC making landfall in Japan^30^. As this event was not included in the reanalysis data used in the prior research^9^, it serves as an extreme case beyond the range of the training data.

## Data

Pix2Pix requires paired input (source) and target images (ground truth). The input images consist of the spatial distributions of zonal and meridional wind speeds, as well as sea level pressure, simulated by a parametric TC model (PM). Best track data (e.g., TC position and central pressure) provided by the RSMC Tokyo via the International Best Track Archive for Climate Stewardship (IBTrACS)^19^ were used as inputs for the PM. These data were linearly interpolated to 1-hour intervals for training, validation, and test datasets, and to 10-minute intervals for the hindcast of TC Faxai. For the target images during pre-training, DSJRA-55^18^ provided by the Japan Meteorological Agency (JMA) was used. DSJRA-55 is a dataset downscaled to a 5 km grid around Japan using the JRA-55 reanalysis^31^ as initial and boundary conditions, covering the 55-year period from 1958 to 2012 with a 1-hour temporal resolution. To focus on TCs approaching the Tokyo Bay area, 44 TCs that passed within a 200 km radius of the reference point (139.5°E, 35.5°N) were selected. Hereinafter, DSJRA-55 is referred to as “reanalysis data.” For fine-tuning, the augmented dataset described later was used.

To evaluate the accuracy of the TC and storm surge simulations, observed wind speeds, wind directions, sea level pressures, and tide levels around Tokyo Bay were utilized. Observed wind speeds were height-corrected to 10 m using the 1/7th power law based on the instrument heights. Tidal residuals were calculated by subtracting astronomical tides (simulated using JMA astronomical arguments) from the observed tide levels. Furthermore, a Lanczos-cosine filter^32^ was applied to the tidal residuals to remove long-period components (filter length: 21 days, cutoff period: 5 days). The locations of observation points are shown in Fig. 1(a), with their coordinates and sampling periods provided in Supplementary Table S1.

## Methods

### Generation of TCs for data augmentation

**Fig. 2:**
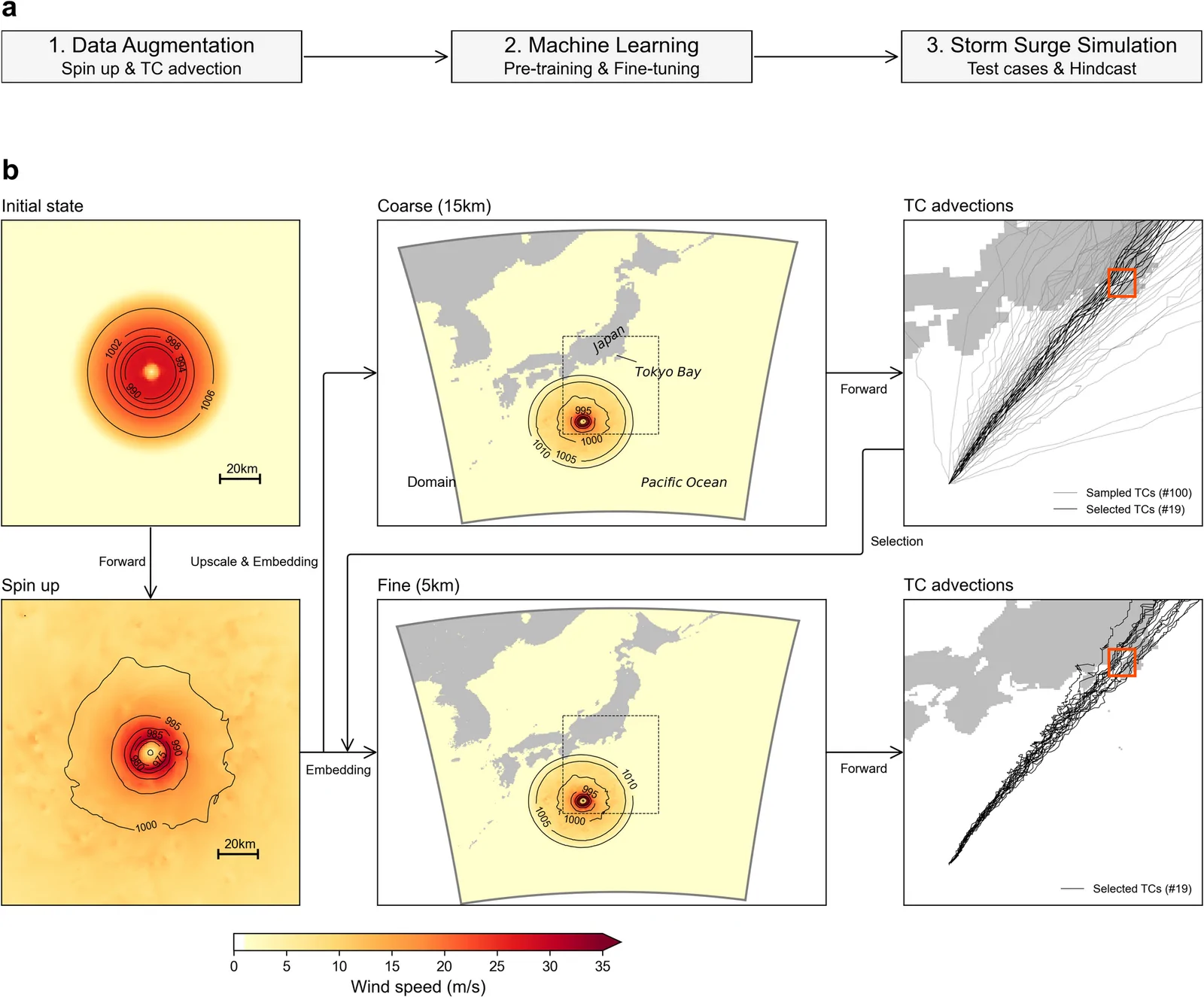
(a) Overall workflow from data augmentation to storm surge simulation. (b) Process of TC spin-up and advection simulations. The center and right columns illustrate the embedding and advection simulations with grid spacings of 15 km (upper row) and 5 km (lower row), respectively. The orange frames in the right column indicate the target area (139.5°E–140.2°E, 35.0°N–35.7°N) used for TC selection.

This study comprises three key elements: data augmentation, machine learning, and storm surge simulation (Fig. 2(a)). The workflow for data augmentation is based on an advection simulation method for TCs within a uniform wind field over realistic topography (i.e., the Hybrid WRF Cyclone Model proposed by^27^) (Fig. 2(b)). First, spin-up simulations were performed over the ocean using the WRF model, utilizing an axisymmetric TC model (RE87)^25^ to make the initial condition (Fig. 2(b), left column). The resulting vortex was then embedded into the computational domain of the reanalysis data (Fig. 2(b), center column). Subsequently, spatially uniform zonal and meridional wind components—representing various large-scale atmospheric flows—were superimposed onto the field. Finally, TC advection under this composite wind field was simulated using WRF (Fig. 2(b), right column). This method physically captures complex topographic effects, such as TC deformation induced by land. Accordingly, it has also been employed to examine the influence of the landfall angle of TCs relative to the coastline on storm surge characteristics^33^. In this study, the simulations were performed using WRF v4.6.1.

### Spin up

Frank’s composite TC soundings in the Northwest Pacific^34^, utilized in the JMA TC bogussing scheme^35^, were used to simulate the initial condition for the spin-up simulations (i.e., 3-dimensional TC fields simulated by RE87). Geopotential heights at specific isobaric surfaces were calculated using the hypsometric equation^36^ to convert the soundings from specific pressure levels to heights. Air temperature was also converted into potential temperature for the simulation. Notably, Frank’s soundings are defined at various radial distances from the TC center. For potential temperature, the azimuthally averaged sounding at each height was applied across all computational grids, as it showed little variation with distance. In contrast, due to the substantial variation of specific humidity with radial distance, the sounding profile observed at 0.7° (the radius closest to the TC center) was utilized throughout the domain to prioritize the accurate representation of physical processes within the TC core.

The first domain was set with a horizontal grid spacing of 15 km and 202 × 202 grid points. The second domain, nested near the TC center, had a grid spacing of 5 km and 202 × 202 grid points. A total of 21 vertical layers were employed. With these settings, a one-week spin-up simulation was performed, with time steps for the first and second domains were set to 60 s and 20 s, respectively. The physics options used were identical with those in the prior study^27^. The parameters in RE87 were defined as follows: the Coriolis parameter was 7.3 × 10⁻⁵ s^− 1^ (equivalent to a latitude of 30°N), the depth of vortex was 20 km, the radius of maximum wind ($\:{r}_{\mathrm{m}}$) was 25 km, the outer radius ($\:{r}_{\mathrm{o}}$) was 200 km, the maximum wind speed ($\:{v}_{\mathrm{m}}$) was 45 m/s, and the sea surface temperature (* SST*) was 28 °C based on preliminary studies (see Supplementary Text 1). The resulting spin-up TC had a maximum wind speed of 43 m/s, a central pressure of 950 hPa, a 50 kt winds radius of 95 km, and a 30 kt winds radius of 202 km. This case was selected as a representative extreme case and used for subsequent advection simulations.

### Advection of TCs

Initial TC positions were set at 30°N latitude, ranging from 135°E to 145°E longitude at 2° intervals. Frequency distributions of TC translation speeds and directions were extracted from the best track data within sampling areas (1° × 1° width) centered on the TC positions. Zonal and meridional wind speeds were then sampled from log-normal distributions fitted to the extracted data and applied uniformly across all computational grids. By applying these wind components across both horizontal and vertical directions, a 3-dimensionally uniform background wind field was established. The TC wind field obtained from the spin-up simulation was then superimposed onto this background field to initialize the advection simulation. While this step enables the numerical simulation of TC advection driven by large-scale atmospheric flow, some background fields caused TCs to deviate from Tokyo Bay. To efficiently generate TCs that approach the bay, the Python library Optuna^37^ was utilized to optimize the background field. In this process, Optuna was utilized to optimize the quantiles of the fitted log-normal distributions. These values were then mapped to specific wind speeds through the inverse cumulative distribution function (CDF). The objective function was defined as the minimum distance between the center of the advected TC and a reference point located at (35.5°N, 139.5°E).

Of note, Parameter optimization via Optuna is computationally expensive, as it requires a WRF simulation for each trial. To reduce computational cost, the spin-up TC was first embedded in the computational domain with a 15 km grid spacing, and a total of 600 advection simulations were performed (i.e., 100 variations for each embedding point). From these, 100 TCs passing through the target area (orange frame in Fig. 2(b)) were selected, and the corresponding background fields were identified. By filtering TCs that pass through the target area, we avoided performing redundant high-resolution simulations on irrelevant cases out of the 600 TCs, thereby reducing the overall computational burden. The background fields were then superimposed onto the TC field embedded in the 5 km grid domain, and advection simulations were performed in the same manner. The results of these 100 cases served as the augmented dataset for machine learning.

During the simulations, surface elevation and land-sea masks from the reanalysis data JRA-3Q^38^ were utilized. For surface temperature, soil temperature, and sea ice fraction, typical summer climatological values were obtained by extracting summer data (July to October) from JRA-3Q monthly averages from January 1991 to December 2024. These values were averaged and spatially interpolated onto the WRF computational grid.

### Pre-training in machine learning

For a total of 44 TCs approaching Tokyo Bay, paired input and ground truth images were created using the PM and the reanalysis data, respectively. The Holland pressure model^39^ was utilized for the PM, with the wind field calculated in a moving coordinate system^40^. For Holland’s scaling parameter * B*, a value of 1 was adopted, as recommended for intense TCs making landfall in Japan^41^. The central pressure and TC position from DSJRA-55 were used as inputs for the PM. The radius of maximum wind, $\:{r}_{\mathrm{m}}$, was determined by minimizing the average difference in pressure fields between the PM and the reanalysis data in the region where sea level pressure was below 995 hPa near the TC center. The wind speed reduction factor, $\:C\left(X\right)$, was calculated using the following formula^42^:

1 $$\:\begin{array}{c}C\left(X\right)=C\left(\infty\:\right)+\left[C\left({X}_{\mathrm{p}}\right)-C\left(\infty\:\right)\right]{\left(\frac{X}{{X}_{\mathrm{p}}}\right)}^{k-1}\cdot\:\exp\left[\left(1-\frac{1}{k}\right)\left\{1-{\left(\frac{X}{{X}_{\mathrm{p}}}\right)}^{k}\right\}\right]\end{array}$$

Here, * X* is the distance from the TC center normalized by $\:{r}_{\mathrm{m}}$ and $\:C\left({X}_{\mathrm{p}}\right)$ is the wind speed reduction factor at $\:X={X}_{\mathrm{p}}$. The model parameters were set to $\:{X}_{\mathrm{p}}=1$, * k=2.5*, and $\:C\left(\infty\:\right)=2/3$. The value of $\:C\left({X}_{\mathrm{p}}\right)$ was determined by minimizing the difference in maximum wind speed between the PM and the reanalysis data. The inflow angle was set to 30 degrees. Following previous studies on wind field downscaling using deep learning^15,16^, a five-channel tensor was constructed as the input image, comprising the spatial distributions of sea level pressure, zonal/meridional wind speeds, surface elevations, and land-sea masks. For the ground truth image, a three-channel tensor was created using the sea level pressure and zonal/meridional wind speeds from the reanalysis data. All tensors had spatial dimensions of 512 ×512 pixels. The dataset consisted of 2,568 snapshots extracted at 1-hour intervals from the 44 TCs. These images were split such that the frequency distribution of central pressure remained consistent across subsets, resulting in 1920 images for training, 268 for validation, and 380 for testing. Each image was normalized channel-wise using the mean value of the entire dataset.

**Fig. 3:**
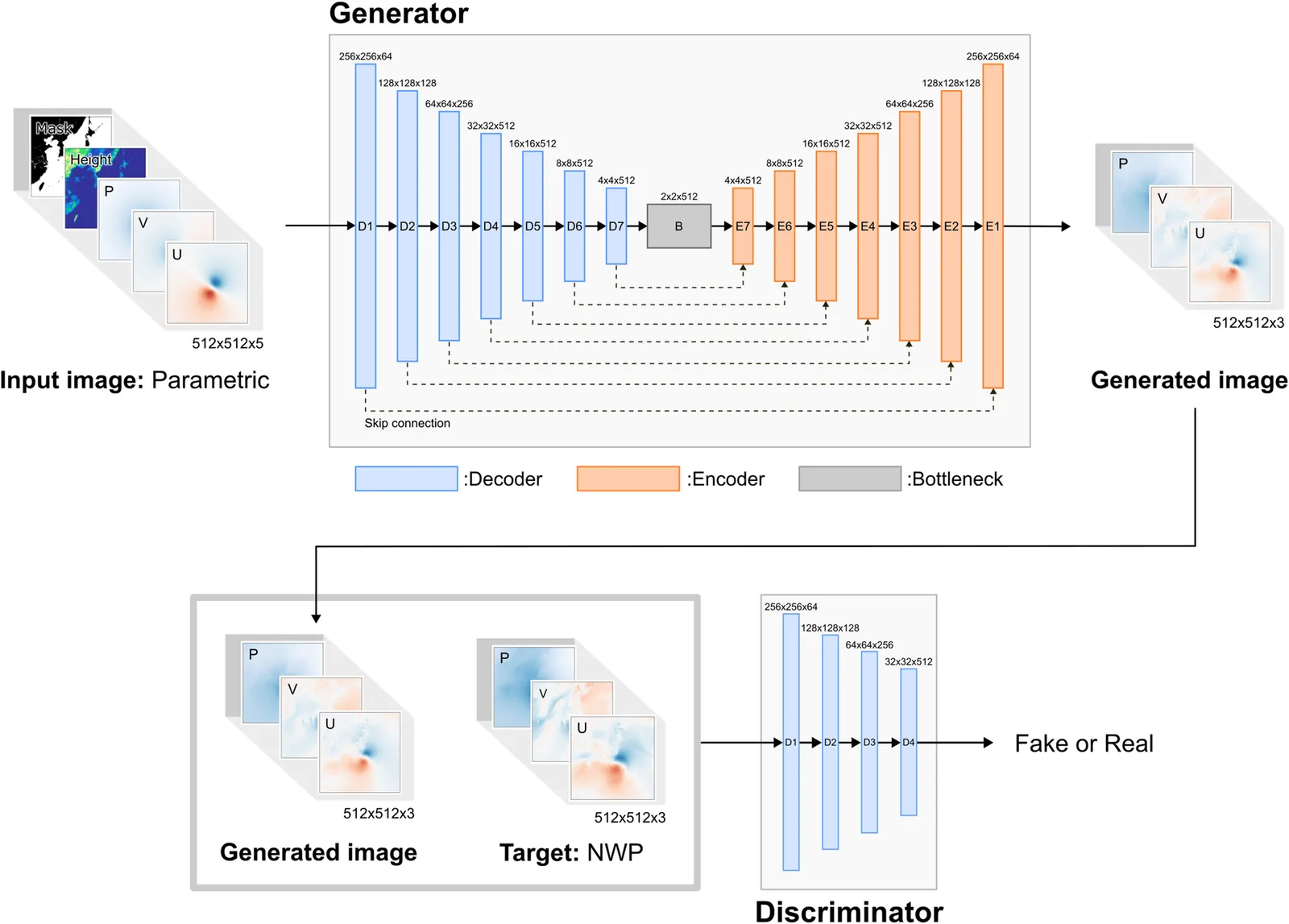
Configuration of the Pix2Pix model. Decoder blocks, encoder blocks, and a bottleneck block are labeled as D, E, and B, respectively. The numbers in the figure indicate the tensor dimensions in terms of rows, columns, and channels.

The architecture of Pix2Pix, illustrated in Fig. 3, is largely consistent with the original implementation^28^ and the previous study^9^. However, the discriminator’s learning rate and the momentum parameter $\:{\beta\:}_{1}$ were set to 0.00001 and 0.1, respectively, to stabilize the training process. Detailed parameters are summarized in Table 1. The model was trained for 200 epochs using an NVIDIA H100 GPU (94 GB).

**Table 1:** Parameter settings used in Pix2Pix.

Parameters	Value
Optimizer	Adam
Learning rate	0.0002 (for Generator) 0.00001 (for Discriminator)
Momentum parameter $\:{\beta\:}_{1}$	0.5 (for Generator) 0.1 (for Discriminator)
Momentum parameter $\:{\beta\:}_{2}$	0.999
Weight of $\:{L}_{1}$ loss	100
Epoch number	200

### Fine-tuning

Fine-tuning was initialized using the model pre-trained for 200 epochs on the reanalysis data of 44 TCs. The augmented dataset consisting of 100 TCs derived from the advection simulations was utilized. One TC was selected for validation and another for testing from each of the six advection starting points, while the remaining 88 TCs were assigned to the training set. Based on 1-hour snapshots from these TCs, the final augmented dataset comprised 1,944 images for training, 141 for validation, and 136 for testing. The model configuration and the methodology for generating the input images were identical to those used during pre-training.

To mitigate catastrophic forgetting^43^, we formulated the generator’s loss by incorporating the loss from the pre-training dataset as a regularization term. This strategy is inspired by regularization-based fine-tuning methods, such as Elastic Weight Consolidation^44^. Specifically, the total loss $\:{\mathcal{L}}_{\mathrm{G}}\left(\boldsymbol{\theta\:}\right)$ was defined as follows:

2 $$\:\begin{array}{c}{\mathcal{L}}_{\mathrm{G}}\left(\boldsymbol{\theta\:}\right)={\mathcal{L}}_{\mathrm{G}}\left({\boldsymbol{\theta\:}}_{\mathrm{D}\mathrm{A}}\right)+\lambda\:{\mathcal{L}}_{\mathrm{G}}\left({\boldsymbol{\theta\:}}_{\mathrm{R}\mathrm{e}\mathrm{a}\mathrm{n}\mathrm{a}\mathrm{l}\mathrm{y}\mathrm{s}\mathrm{i}\mathrm{s}}\right)\end{array}$$

$\:{\mathcal{L}}_{\mathrm{G}}\left({\boldsymbol{\theta\:}}_{\mathrm{D}\mathrm{A}}\right)$ represents the generator loss for the augmented dataset, while $\:{\mathcal{L}}_{\mathrm{G}}\left({\boldsymbol{\theta\:}}_{\mathrm{R}\mathrm{e}\mathrm{a}\mathrm{n}\mathrm{a}\mathrm{l}\mathrm{y}\mathrm{s}\mathrm{i}\mathrm{s}}\right)$ denotes the loss for the reanalysis data used during pre-training. The term $\:{\lambda\:}$ is a dynamic and norm-based weight^45^ for the pre-training dataset. For the $\:{\mathrm{n}}^{th}\:$image in a certain epoch, the loss function $\:{\mathcal{L}}_{\mathrm{G}}\left({\boldsymbol{\theta\:}}^{\left(\mathrm{n}\right)}\right)$ is calculated as follows:

3 $$\:\begin{array}{c}{\mathcal{L}}_{\mathrm{G}}\left({\boldsymbol{\theta\:}}^{\left(\mathrm{n}\right)}\right)={\mathcal{L}}_{\mathrm{G}}\left({\boldsymbol{\theta\:}}_{\mathrm{D}\mathrm{A}}^{\left(\mathrm{n}\right)}\right)+{{\lambda\:}}^{\left(\mathrm{n}\right)}{\mathcal{L}}_{\mathrm{G}}\left({\boldsymbol{\theta\:}}_{\mathrm{R}\mathrm{e}\mathrm{a}\mathrm{n}\mathrm{a}\mathrm{l}\mathrm{y}\mathrm{s}\mathrm{i}\mathrm{s}}^{\left(\mathrm{n}\right)}\right)\end{array}$$

Here, $\:{{\lambda\:}}^{\left(\mathrm{n}\right)}$ is calculated as:

4 $$\:\begin{array}{c}{{\lambda\:}}^{\left(\mathrm{n}\right)}=\beta\:{{\lambda\:}}^{\left(\mathrm{n}-1\right)}+\left(1-{\beta\:}\right){\widehat{{\lambda\:}}}^{\left(\mathrm{n}\right)}\end{array}$$

5 $$\:\begin{array}{c}{\widehat{{\lambda\:}}}^{\left(\mathrm{n}\right)}=\frac{\parallel{\nabla\:}_{\boldsymbol{\theta\:}}\:{\mathcal{L}}_{\mathrm{G}}\left({\boldsymbol{\theta\:}}_{\mathrm{D}\mathrm{A}}^{\left(\mathrm{n}\right)}\right)\parallel}{\parallel{\nabla\:}_{\boldsymbol{\theta\:}}\:{\mathcal{L}}_{\mathrm{G}}\left({\boldsymbol{\theta\:}}_{\mathrm{R}\mathrm{e}\mathrm{a}\mathrm{n}\mathrm{a}\mathrm{l}\mathrm{y}\mathrm{s}\mathrm{i}\mathrm{s}}^{\left(\mathrm{n}\right)}\right)\parallel}\end{array}$$

and $\:{\beta\:}$ was set to 0.9. By updating $\:{{\lambda\:}}^{\left(\mathrm{n}\right)}$, the model maintains a balance between the loss on the augmented dataset and that on the reanalysis data. Notably, the number of images in the two datasets differed; therefore, we performed random sampling from the reanalysis dataset at each epoch to create a subset of equal size to the augmented dataset. This subset was then used for calculating the loss. The discriminator’s loss function was defined as the average loss of both the augmented and reanalysis datasets. This follows a prior study^46^, which suggests that the discriminator does not require specific modifications during fine-tuning. To enhance computational efficiency, weight updates were restricted to specific blocks within the Pix2Pix architecture. These target blocks were identified based on the validation process (see Supplementary Text 2). The model was fine-tuned for a total of 200 epochs.

### Storm Surge Modelling

The Regional Ocean Modelling System (ROMS)^47^ was used for storm surge simulations. The computational domain and settings were identical to those used in the previous study conducting storm surge hindcasts in Tokyo Bay^8^. During the simulations, spatiotemporal variations of sea level pressure and wind stress were applied as external forcing; however, the effects of waves and astronomical tides were excluded to focus on the impact of fine-tuning on the storm surges.

In this study, the model performance was evaluated under two different scenarios: a hypothetical scenario using test data from the augmented dataset and a hindcast of a historical extreme event. For the hypothetical scenario, 4 types of external forcing were utilized: the NWP used to create the ground truth images, the PM generated from TC statistics in the NWP, and the PMs converted by the pre-trained and fine-tuned models. Time series of sea levels were extracted at 6 tide gauges in Tokyo Bay (see Fig. 1(a)), and the results from the PM, pre-trained model, and fine-tuned model were compared against the NWP results.

Additionally, a hindcast of the storm surge caused by TC Faxai (2019) was conducted to demonstrate the impact of data augmentation on extreme cases. In this simulation, the PM and its converted results by the pre-trained and fine-tuned models were utilized as external forcing. The TC position and central pressure for the PM were obtained from the best track data^19^. The radius of maximum wind was calculated using an empirical formula^48^, based on the average of the radii of 50 kt winds along the major and minor axes from the best track. The parameter $\:C\left({X}_{\mathrm{p}}\right)$ in Eq. 1 was defined as the ratio of the maximum sustained wind speed in the best track to the maximum gradient wind speed in the PM. This adjustment ensured that the maximum wind speed of the PM (i.e., the maximum at $\:X={X}_{\mathrm{p}}$) matched that of the best track.

## Results and Discussion

### Generation of Physics-based Augmented TCs

**Fig. 4:**
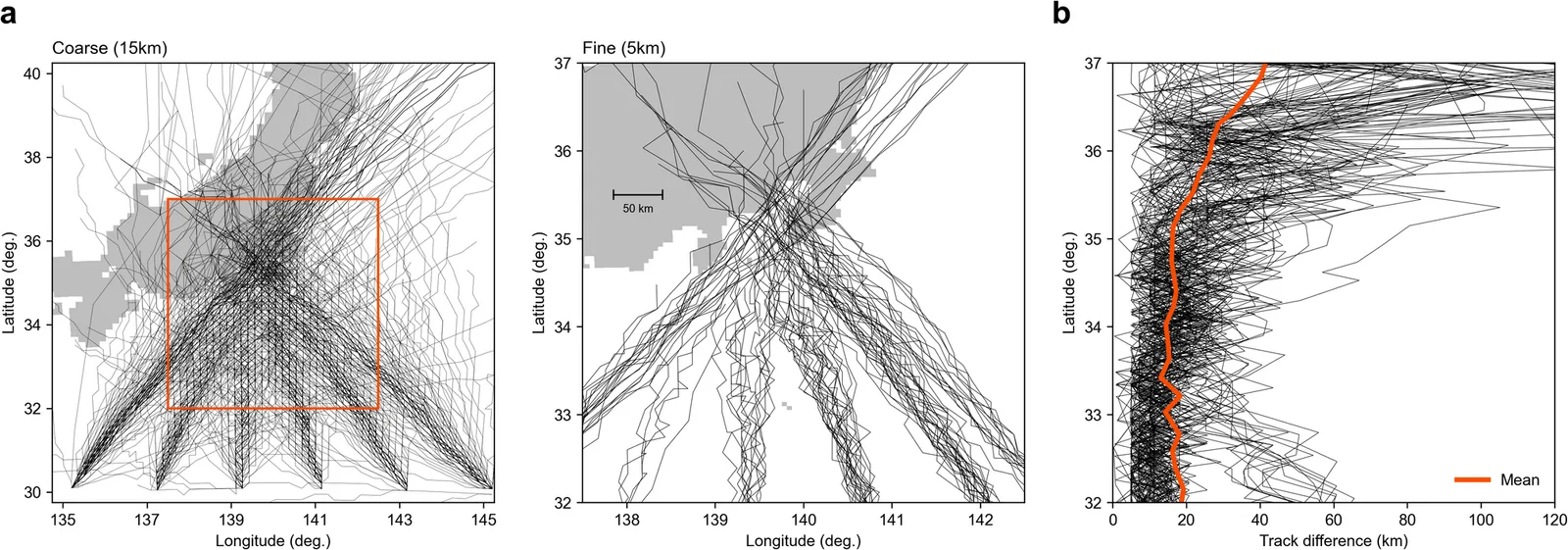
TC tracks obtained from the advection simulations. (a) Thin black lines represent the tracks of all 600 TCs, while thick black lines indicate the tracks of the selected 100 TCs. The left panel shows results for the 15 km grid domain, with the orange rectangle indicating the spatial extent of the middle panel. The middle panel shows results for the 5 km grid spacing. (b) Track differences between TCs simulated using the 15 km and 5 km grid domains. The orange line indicates the mean difference for the 100 selected TCs.

First, the robustness of selecting TCs used in the augmented dataset was evaluated by comparing TC tracks simulated in the 15 km and 5 km domains. As shown in Fig. 4(a), numerous TCs approaching Tokyo Bay were successfully simulated using WRF and Optuna with the 15 km grid spacing. Based on these results, 100 TCs approaching the vicinity of Tokyo Bay were selected, and simulations were re-run with a 5 km grid spacing under identical background wind fields. The discrepancies in TC tracks between the 15 km and 5 km grid domains generally remained within 20 km south of 35°N (Fig. 4(b)). While the track difference increased as the TCs moved northward, the mean difference remained below 30 km even between 35°N and 36°N, the range where Tokyo Bay is located.

**Fig. 5:**
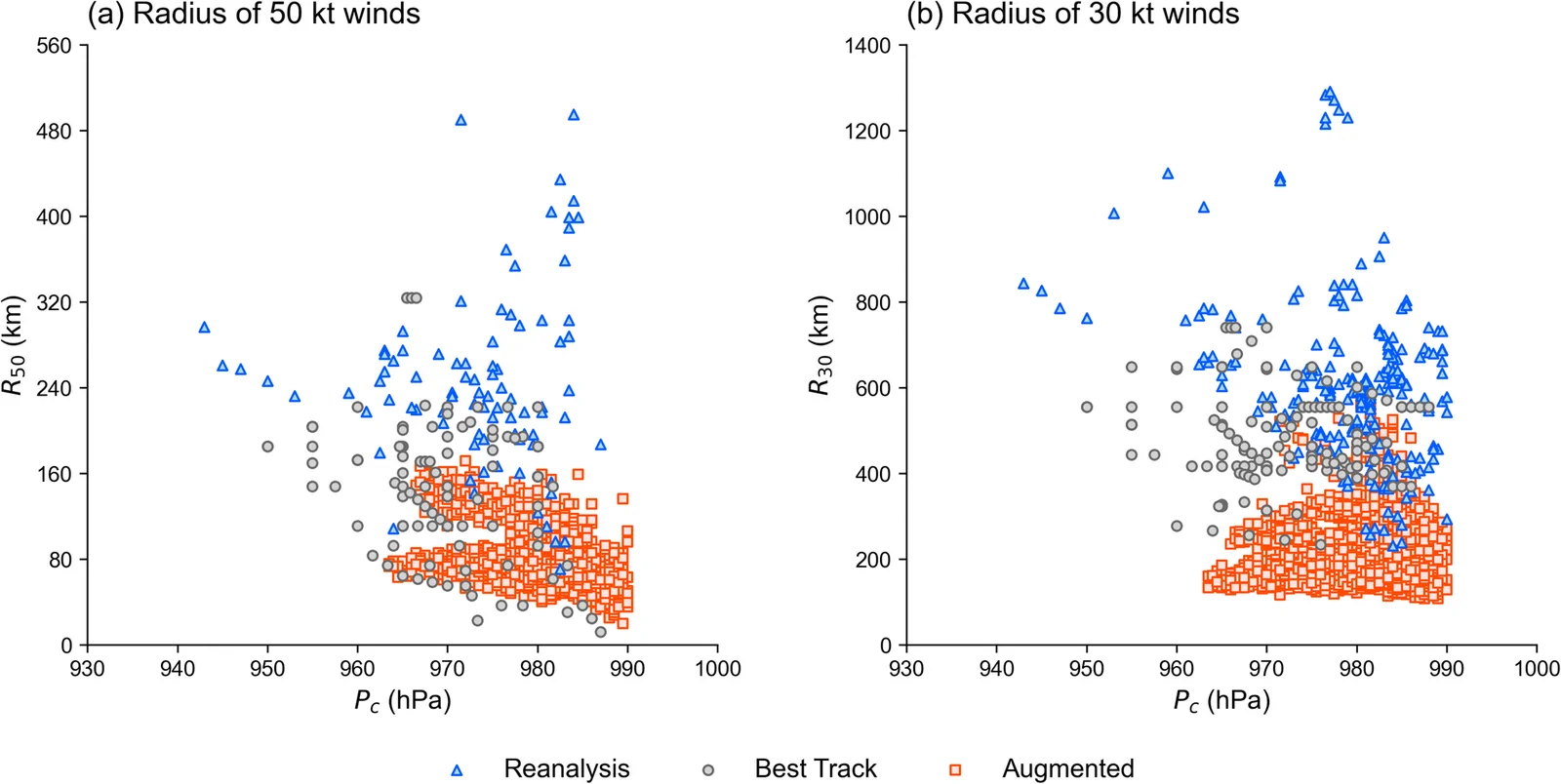
Relationship between central pressure and the radii of 50 kt and 30 kt winds. Definitions of “Reanalysis” and “Best Track” are the same as in Fig. 1b; “Augmented” indicates the results from the advection simulations. The larger sample size in the augmented dataset results from applying the same extraction criteria as in Fig. 1 to all available simulation snapshots.

Next, we generated scatter plots comparing central pressure and the radii of 50 kt and 30 kt winds for the simulated TCs to evaluate the extent to which the TC advection simulations successfully augmented the reanalysis data. Figure 5 compares these results with those of the reanalysis data and the best track. Notably, the augmented dataset covered regions in the scatter plots that were not represented in the reanalysis data. Specifically, numerous TCs with the radius of 50 kt winds below 80 km and the radius of 30 kt winds below 200 km were generated, with the minimum central pressure of nearly 965 hPa.

### Effects of Data Augmentation on TC fields

**Fig. 6:**
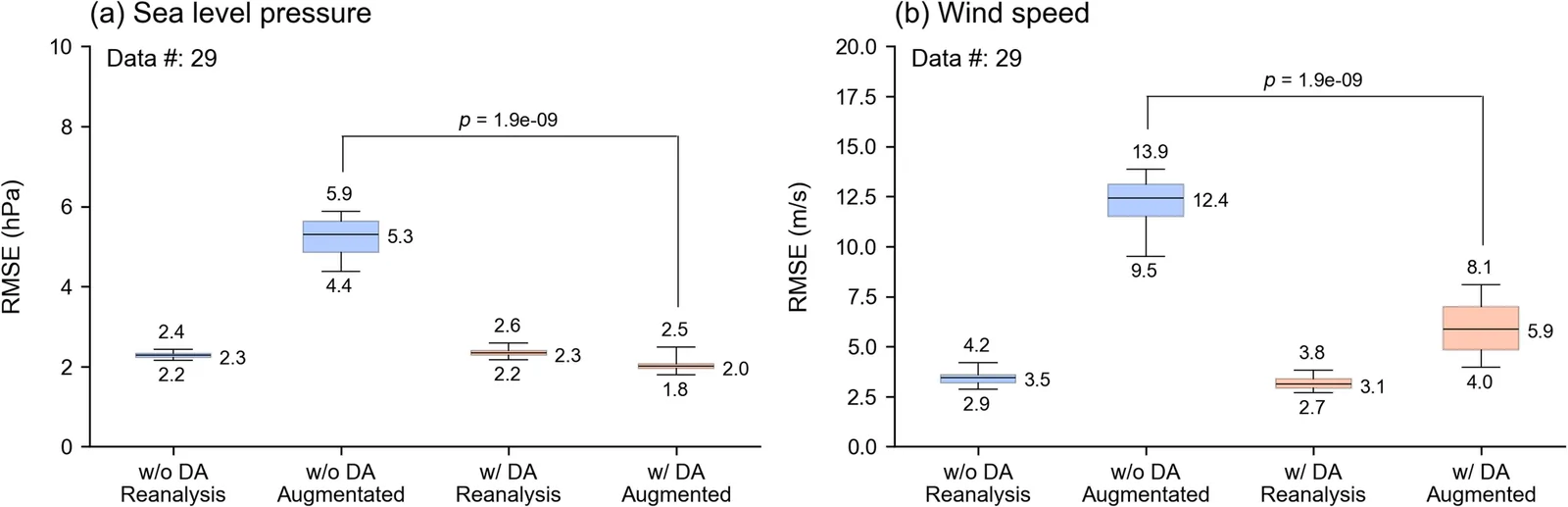
Box plots of RMSE for sea level pressure and 10 m wind speed in the test data. RMSE calculated across all 29 grid points within Tokyo Bay is categorized by the presence of fine-tuning (w/o DA and w/DA) and the type of test data (Reanalysis and Augmented). Numerical values at the top, middle, and bottom of each box indicate the maximum, median, and minimum RMSE, respectively. * p*-values comparing w/o DA and w/DA in the augmented dataset were calculated using the Wilcoxon signed-rank test.

The impact of fine-tuning on the TC conversion by Pix2Pix was evaluated using a model trained for 100 epochs, which achieved the highest accuracy on the validation data (see Supplementary Text 3). RMSE was calculated by comparing the converted results against the time series of sea level pressure and 10 m wind speed from the test data at all grid points within Tokyo Bay. For the reanalysis data used in pre-training, the pre-trained model (w/o Data Augmentation (DA)) and the fine-tuned model (w/DA) demonstrated relatively similar performance (Fig. 6). Specifically, the median RMSE values for sea level pressure and 10 m wind speed were 2.3 hPa and 3.5 m/s for the pre-trained model, and 2.3 hPa and 3.1 m/s for the fine-tuned model, respectively. In contrast, for the augmented dataset, the pre-trained model (w/o DA) exhibited higher errors, with median RMSE values of 5.3 hPa for sea level pressure and 12.4 m/s for 10 m wind speed. By applying fine-tuning (w/DA), these errors were reduced to 2.0 hPa and 5.9 m/s, respectively. The * p*-values comparing the w/o DA and w/DA cases further confirm the statistical robustness of these improvements. These results indicate that fine-tuning significantly enhanced the conversion performance for the PM within the augmented test data.

**Fig. 7:**
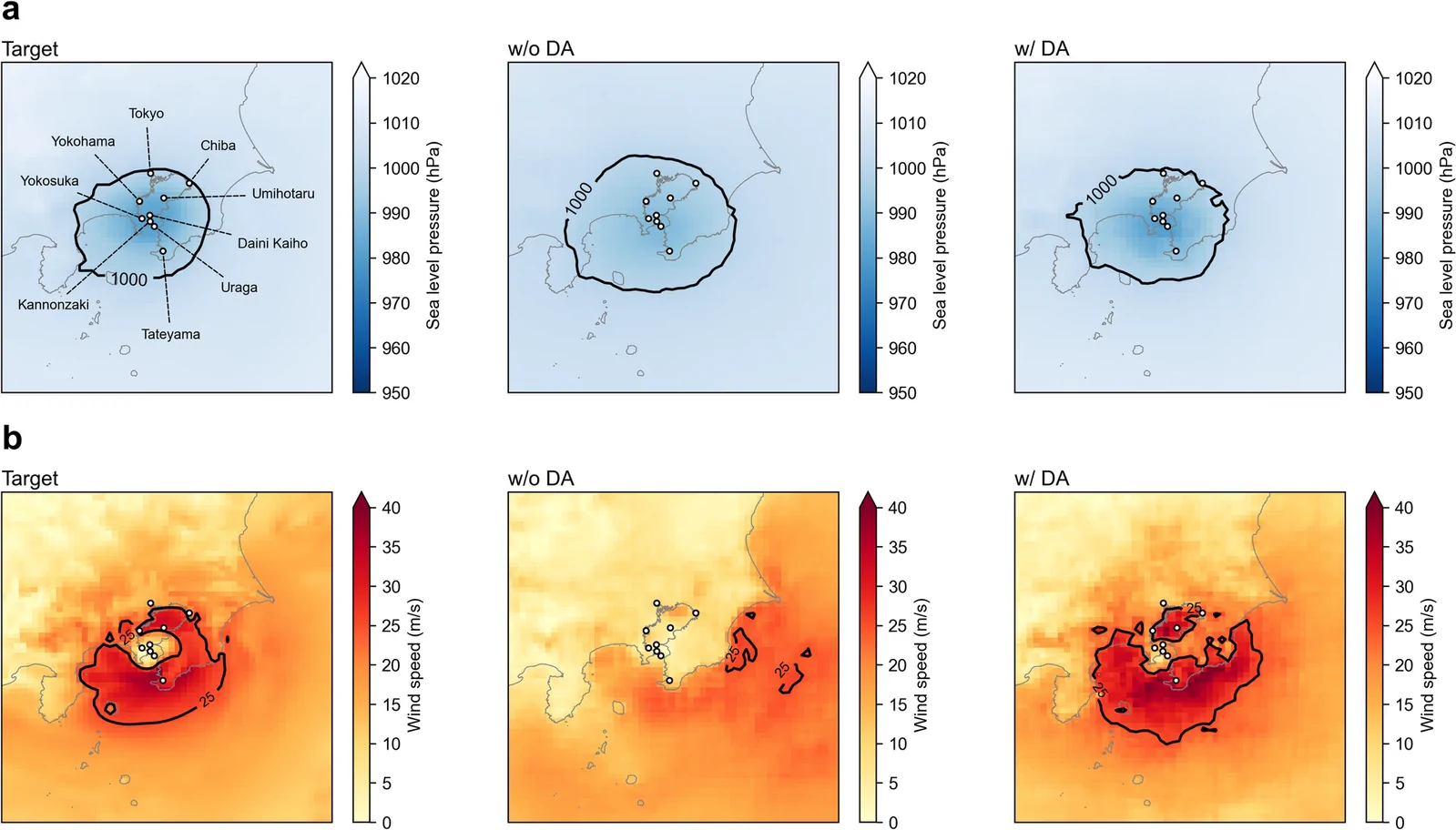
Comparison of TC meteorological fields: (a) sea level pressure and (b) 10 m wind speed. “Target” indicates the ground truth image (NWP) from the augmented dataset; “w/o DA” and “w/DA” denote the result of PM conversion using the pre-trained and the fine-tuned models, respectively. White dots show the locations of weather station around Tokyo Bay. Detailed locations are provided in Fig. 1(a).

These improvements were further confirmed by visualizing the converted TC fields. Figure 7 illustrates the results of applying the pre-trained and fine-tuned models to the PM pressure and wind fields in the augmented test data. Regarding the pressure field (Fig. 7(a)), the extent of the 1000 hPa isobar showed no marked difference between the models; however, the central pressure was slightly higher in the pre-trained model (w/o DA) compared to the ground truth (Target). Conversely, the fine-tuned model (w/DA) achieved the central pressure closer to the ground truth. Regarding the wind field (Fig. 7(b)), the spatial distributions differed significantly. The pre-trained model showed a substantial decrease in wind speed compared to the ground truth, with the area of wind speeds exceeding 25 m/s almost vanishing. In contrast, the fine-tuned model produced a wind field comparable to the ground truth, successfully maintaining both the intensity and spatial scale of a compact TC.

**Fig. 8:**
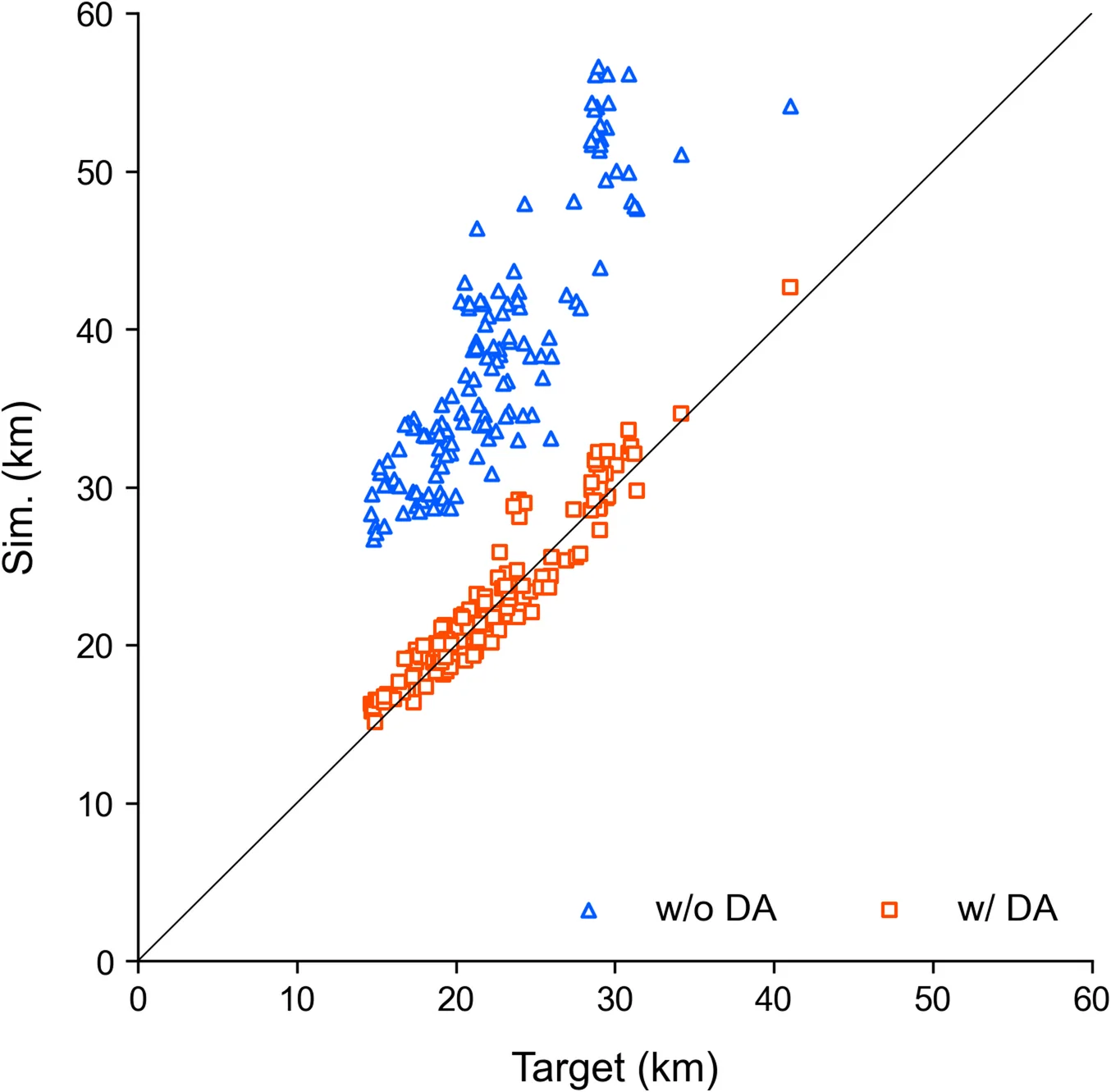
Radius of maximum wind speed in the test data for the w/o DA and w/DA models compared against the ground truth (Target). The definitions of “Target”, “w/o DA”, and “w/DA” are the same as those in Fig. 7.

Data augmentation also has a substantial impact on the spatial scale of the converted TC. We estimated the radius of maximum wind speed $\:{r}_{0}$ of the TCs in the ground truth, pre-trained, and fine-tuned models using the following formula^48^:

6 $$\:\begin{array}{c}{r}_{0}=r\:\ln\frac{{P}_{0}-{P}_{\mathrm{c}}}{P\left(r\right)-{P}_{\mathrm{c}}}\end{array}$$

where * r* is the distance from the TC center, $\:{P}_{0}$ is the environmental pressure (1013.25 hPa), $\:{P}_{\mathrm{c}}$ is the central pressure, and $\:P\left(r\right)$ is the atmospheric pressure at a given distance * r*. Here, $\:{r}_{0}$ was calculated by setting * r* as the distance to the 1000 hPa isobar. Notably, the fine-tuned model yields more accurate and smaller $\:{r}_{0}$ values than the pre-trained model when applied to the augmented dataset (Fig. 8). This result demonstrates that fine-tuning effectively enhances the representation of TC spatial scales, particularly for compact and intense systems.

Consequently, it is evident that for TCs located beyond the range of reanalysis data, the conversion performance of Pix2Pix trained solely on the reanalysis data can degrade. By implementing physics-based data augmentation and fine-tuning, the conversion performance is significantly improved even for extreme TCs.

### Storm surge simulations in Test Data

**Fig. 9:**
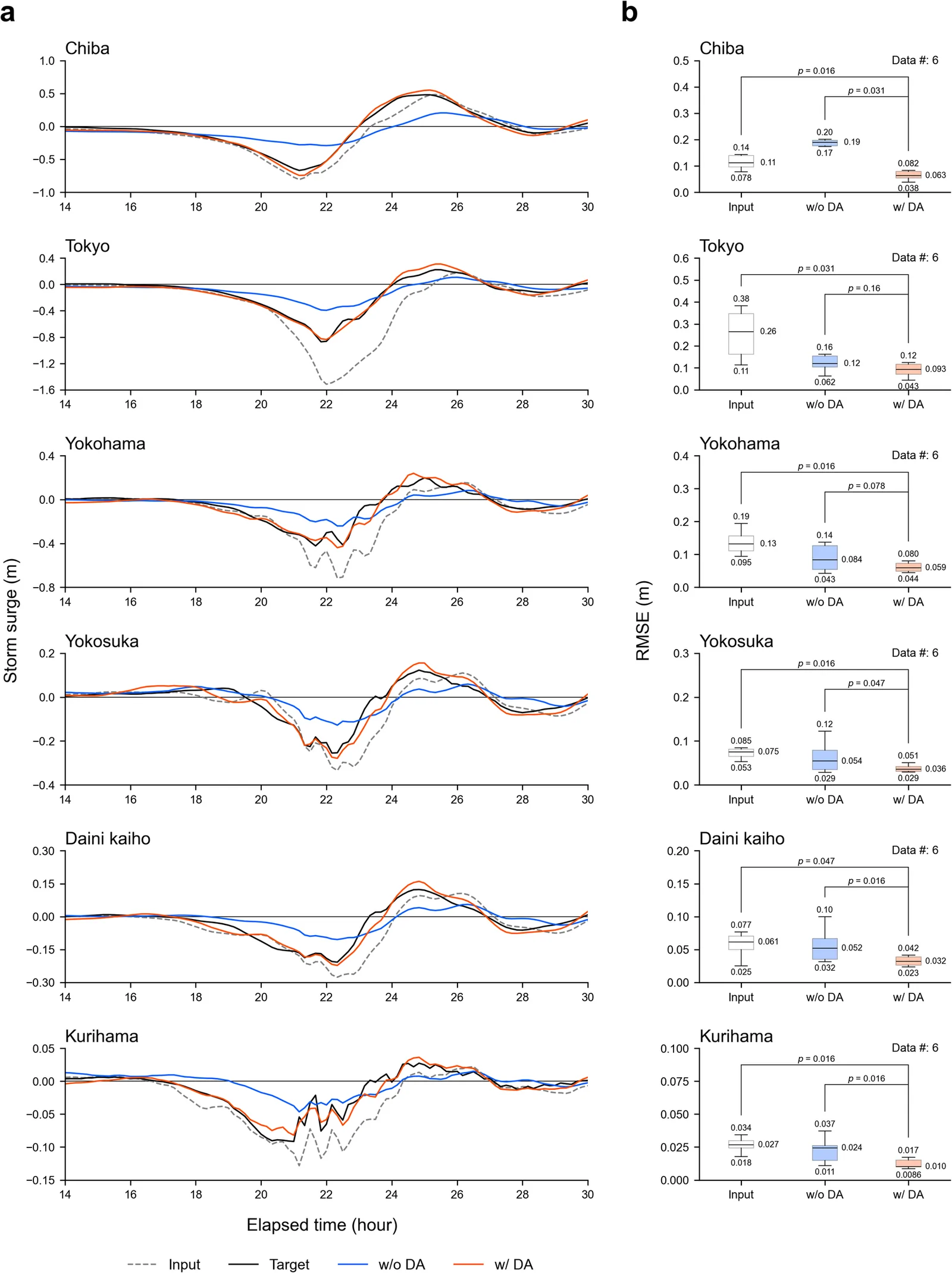
(a) Time series of storm surges for a TC advected from (137E, 30 N). Results are shown for the PM used as the Pix2Pix input (Input), the NWP as the ground truth (Target), the pre-trained model (w/o DA), and the fine-tuned model (w/DA). (b) Box plots of storm surge RMSE for all 6 TCs in the test data. Numerical values at the top, middle, and bottom of each box indicate the maximum, median, and minimum RMSE, respectively. * p*-values comparing the PM vs. the fine-tuned model and the pre-trained model vs. the fine-tuned model were calculated using the Wilcoxon signed-rank test.

Storm surges were simulated for the 6 TCs in the augmented test dataset using 4 types of external forcing: the NWP (ground truth), the PM (Pix2Pix input), and the PM converted by the pre-trained and fine-tuned models. The resulting time series of sea levels was compared at the 6 tide gauges in Tokyo Bay. Figure 9(a) illustrates the time series for a TC advected from (137°E, 30°N). Compared to the ground truth (Target), the PM (Input) showed an excessive drop in simulated storm surges, particularly at Tokyo, whereas the pre-trained model (w/o DA) generally underestimated the surge amplitude. In contrast, the fine-tuned model (w/DA) yielded results that closely matched the ground truth. Additionally, the PM exhibited the highest RMSE across all 6 test TCs, reaching its value of approximately 0.38 m at Tokyo (Fig. 9(b)). Although the pre-trained model mitigated the average discrepancy relative to the PM, its maximum RMSE occasionally exceeded that of the PM; for instance, at Chiba, the pre-trained model reached 0.20 m, whereas the PM reached 0.14 m. The fine-tuned model achieved the lowest RMSE at all tide gauges, with a maximum of only 0.12 m at Tokyo. These findings demonstrate that fine-tuning significantly improves the accuracy of storm surge simulations for TCs beyond the range of the reanalysis data. Statistical significance between the PM, pre-trained, and fine-tuned models was evaluated at each tide gauge using the Wilcoxon signed-rank test (* n* = 6). Although the fine-tuned model yielded relatively higher * p*-values at Tokyo (0.16) and Yokohama (0.078), the median RMSEs at these stations decreased compared to the pre-trained model. At all other tide gauges, * p*-values were below 0.05. These results indicate that data augmentation generally improves storm surge simulation accuracy.

### Storm surge simulations in extreme case: TC Faxai

**Fig. 10:**
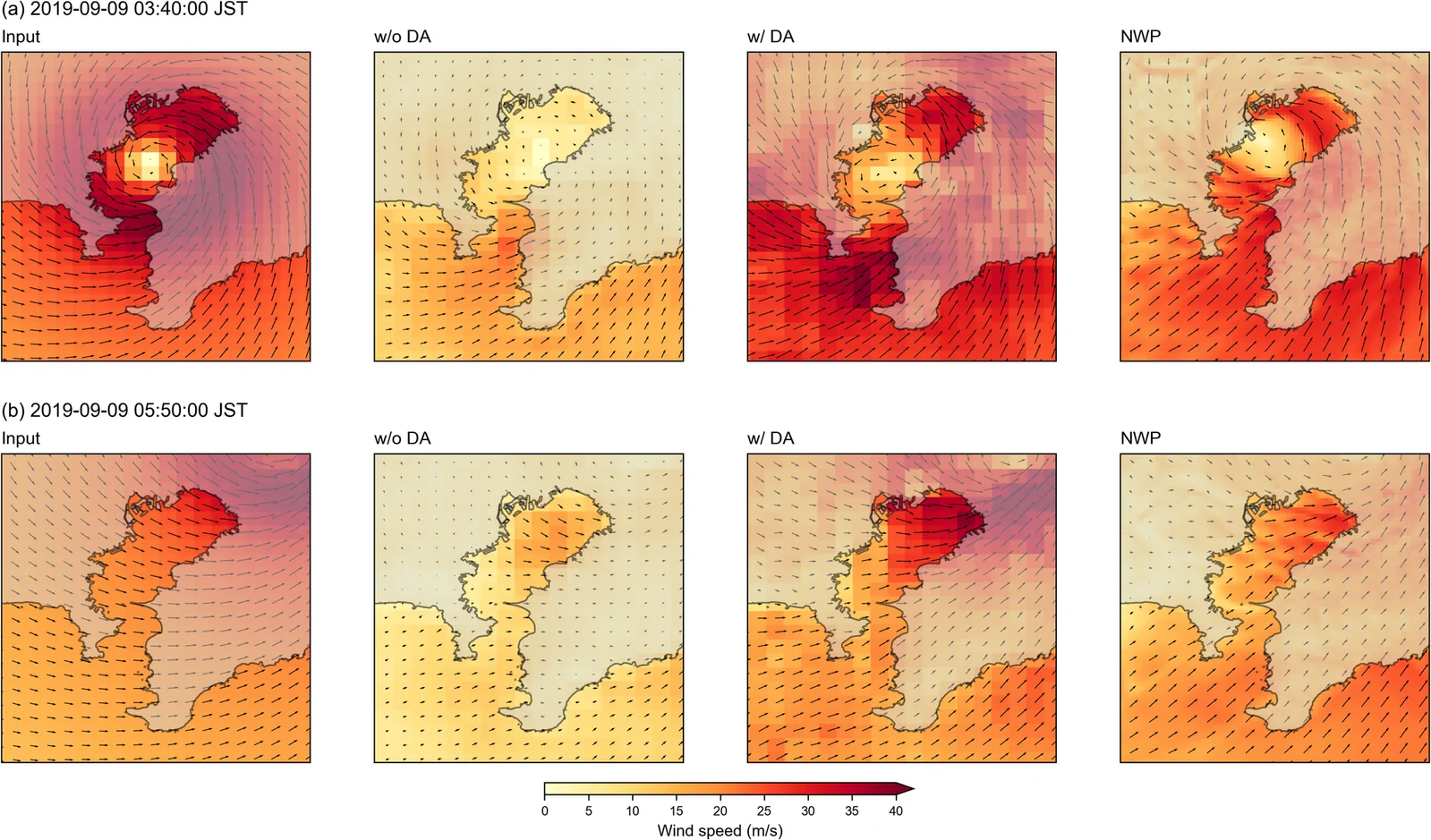
Comparison of wind fields around Tokyo Bay during TC Faxai. (a) During passage. Results obtained by the PM used as the Pix2Pix input image (Input), the pre-trained model (w/o DA), and the fine-tuned model (w/DA) are shown. The rightmost panel shows the result of downscaling analysis values provided by JMA to a 1 km grid width using WRF^8^. (b) During landfall. Label definitions are identical to (a).

The wind fields during TC Faxai’s landfall were examined for the PM, the pre-trained model, and the fine-tuned model. For comparison, the downscaled result of JMA’s Meso-Scale Model (MSM) using WRF^8^ was also depicted. As shown in Fig. 10(a), the PM neglects the influence of land topography on the wind field, resulting in a concentric wind speed distribution even during the passage of TC Faxai. In the pre-trained model (w/o DA), while the wind speed contrast due to topographic effects emerged, an excessive decrease in wind speed relative to the PM occurred simultaneously. In contrast, the fine-tuned model (w/DA) successfully produced wind speeds comparable to the PM while incorporating realistic topographic contrast. Additionally, at the time after the landfall (Fig. 10(b)), the fine-tuned model simulated wind directions over the sea predominantly from the east and northeast, which contributes to the wind setup at the inner bay. These simulated wind directions align closely with the WRF downscaling result. Notably, the fine-tuned model successfully captured the temporal evolutions of sea level pressure and 10 m wind speeds observed around Tokyo Bay (see Supplementary Figs. S5 and S6).

**Fig. 11:**
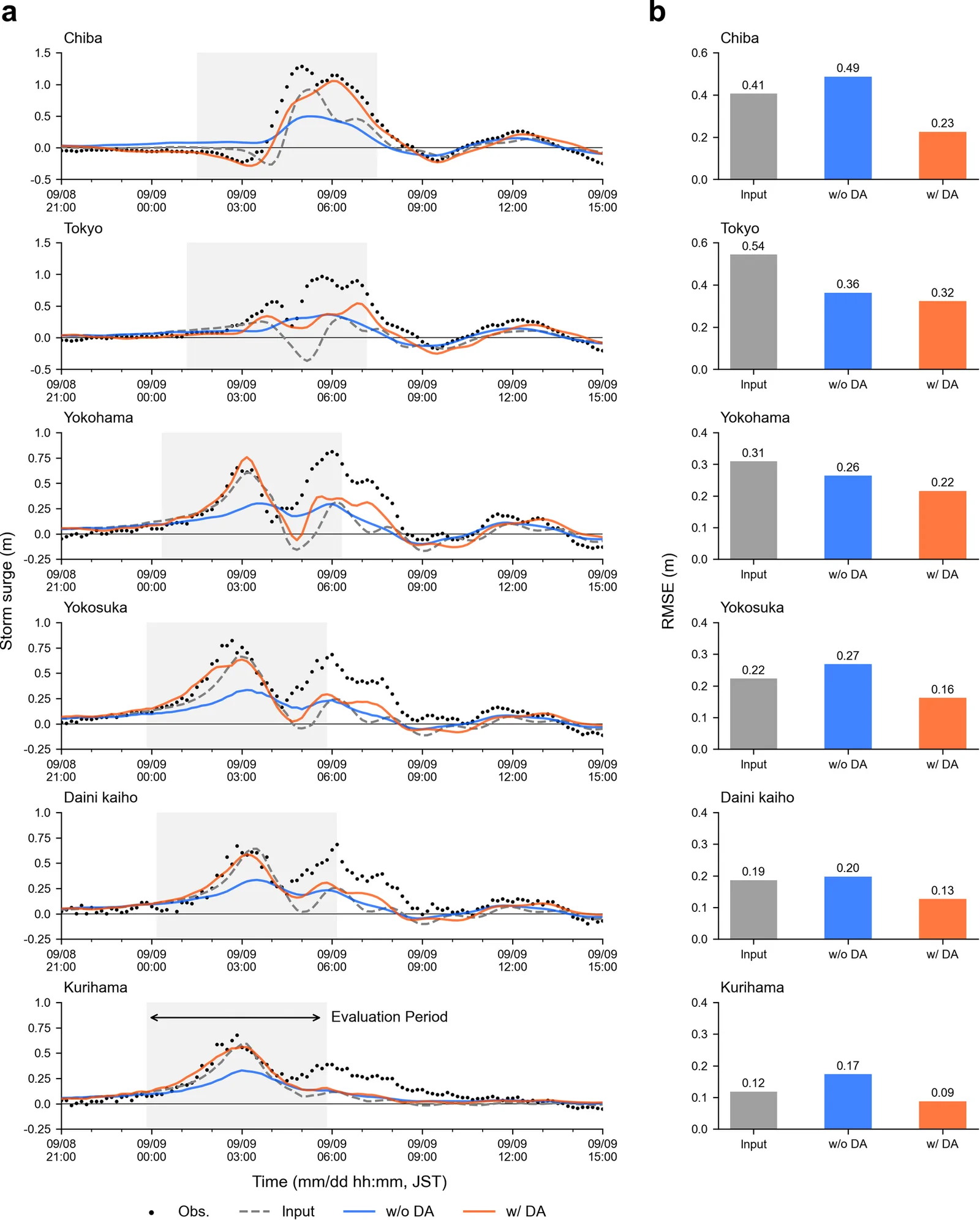
Comparison of storm surge hindcast results for TC Faxai. (a) Results obtained by the PM used as the Pix2Pix input image (Input), the pre-trained model (w/o DA), and the fine-tuned (w/DA) are shown. “Obs.” indicates observed values. (b) RMSE of water level time series against observed values. RMSE was calculated for the 3 h before and after the time the TC approached the tide gauge closest (hatched section in Fig. 11(a)).

Next, the results of the storm surge hindcast for TC Faxai are compared (Fig. 11). Around 3:00 AM on the 9th, as the TC passed near the Miura Peninsula (Fig. 1(a)), local peaks of storm surge exceeding 0.5 m occurred at Daini Kaiho, Kurihama, Yokohama, and Yokosuka (Fig. 11(a)). The PM reproduced these first peaks with an accuracy within 0.2 m error (Input); however, the pre-trained model (w/o DA) resulted in an overall underestimation of up to 0.5 m at Yokosuka. In contrast, the fine-tuned model (w/DA) successfully simulated the peaks with an accuracy nearly equivalent to that of the PM. During the passage of TC Faxai over the bay, local peaks around 4:00 AM and 5:00 AM were observed at Tokyo and Chiba, respectively. The pre-trained model underestimated these peaks, and the PM exhibited particularly low accuracy at Tokyo, resulting in an excessive drop of nearly 0.5 m at the time of the observed 1.0 m peak. In contrast, the fine-tuned model successfully mitigated the drop seen in the PM and achieved closer alignments with the observations than the pre-trained model. This improvement can be attributed to the refined wind fields: during this period, the wind field of the fine-tuned model closely resembled the NWP, whereas the pre-trained model significantly underestimated the wind speeds. After the landfall (around 5:50 AM), the fine-tuned model also improved the accuracy of storm surges at Chiba, increasing the second peak by approximately 0.6 m compared to the pre-trained model. During this phase, southwesterly winds contributing to wind setup were dominant, and only the fine-tuned model could accurately reproduce this wind field (see Fig. 10(b) and Supplementary Fig. S5). In contrast, the wind direction in the PM was northwest, and the pre-trained model simulated remarkably low wind speeds. These localized improvements are clearly reflected in the RMSE calculated between the observed and simulated storm surges (Fig. 11(b)). The fine-tuned model maintained high consistency across all stations, with RMSE values ranging from a minimum of 0.09 m to a maximum of 0.32 m. In contrast, the PM and the pre-trained model exhibited significantly larger discrepancies, with RMSE values ranging from 0.12 m to 0.54 m and 0.17 m to 0.49 m, respectively.

Of note, for storm surges occurring after 6:00 AM, even the fine-tuned model failed to capture the secondary peaks with high accuracy, except in Chiba. Given that the sea level pressure at nearby weather stations had recovered to approximately 1000 hPa and the 10 m wind speed had decreased to roughly 20 m/s (see Supplementary Figs. S5 and S6), the errors seen after the landfall are likely due to physical processes excluded from the storm surge model used (e.g., wave setup), rather than inaccuracies in the external forcings. Improving the reproducibility of these peaks remains a challenge for future research.

### Applicability and limitations

The primary contribution of this study is the establishment of a physics-based data augmentation framework applicable to TC and storm surge modelling. We also show that the accuracy of TC intensity in the deep learning model Pix2Pix was improved by using the augmented dataset generated by the proposed framework. This improvement can be attributed to both the refined representation of TC scales and the topographic effects on TC wind fields. In the pre-trained model, wind speeds and pressure drops for intense, compact TCs were underestimated because such cases were underrepresented in the reanalysis dataset. By fine-tuning with the augmented dataset, the model successfully learned to reproduce the sharp gradients and peak intensities characteristic of compact TCs.

Notably, the proposed framework is applicable to any coastal region, provided that a computational domain can be configured in WRF to simulate the computational grids (or a subset thereof) of reanalysis data, such as DSJRA-55. Our approach is based on the Hybrid WRF Cyclone Model method, which has been successfully utilized for risk assessments of TCs and storm surges along the eastern coasts of Australia^27^ and the United States^33^. This underscores that the fundamental logic of using WRF to generate physically consistent synthetic TCs is a robust strategy for overcoming the scarcity of extreme TC events in reanalysis datasets.

Despite the broad applicability of our framework, certain limitations exist regarding the direct use of the currently trained model. In this study, the Pix2Pix model was trained specifically on TCs affecting the Tokyo Bay area. Therefore, it learned the interactions between the atmosphere and the unique geographic features of the region, such as the topographic effects on TC wind fields induced by the surrounding mountains and urbanized areas. Consequently, the direct application of this specific model to other coastal areas may present challenges in maintaining prediction accuracy without further adaptation. To address this issue, the training dataset should be expanded to include a wider variety of TCs making landfall across broader coastal zones.

Furthermore, while the fine-tuned model successfully demonstrated the ability to transform the parametric TC model (PM) into realistic wind fields while maintaining TC intensity (e.g., for TC Faxai), it is important to note the constraints of spatial resolution. Although the 5 km grid used in this study successfully captured the topographic contrast in wind speed and direction that the PM ignores, this resolution may be coarse for resolving TC fields near the core and local-scale surge amplifications in complex coastal areas. Therefore, validating this framework with higher-resolution NWP data remains an important task for future research to further refine the accuracy of TC and storm surge modelling.

## Conclusions

This study proposed a method to physically augment reanalysis data to improve the diversity of TC training data for deep learning. We fine-tuned the Pix2Pix model using this augmented dataset and validated its impact on TCs beyond the range of reanalysis data, including the actual extreme event of TC Faxai (2019). The main results are summarized as follows:

- To create the augmented dataset, the computational domain of WRF was configured to resemble that of DSJRA-55 with a coarse grid spacing (15 km). TCs after spin-up simulations using the RE87 model were embedded into this domain. TC advection simulations were then performed under various background wind fields, using Optuna to search for zonal and meridional wind components to efficiently select TCs approaching Tokyo Bay. High-resolution simulations (5 km grid spacing) of 100 selected TCs revealed that track differences remained within 20 km relative to the coarse grid results, confirming the robustness of the selection process.
- Fine-tuning was performed with the augmented dataset on the pre-trained Pix2Pix model with DSJRA-55. For test data from the reanalysis, the models before and after fine-tuning showed equivalent accuracy. However, for the augmented test data, the median RMSE for 10 m wind speed over Tokyo Bay decreased from 12.4 m/s (pre-trained) to 5.9 m/s (fine-tuned).
- Storm surge simulations were conducted in Tokyo Bay for 6 TCs in the augmented test dataset, utilizing 4 types of external forcings: the PM (input for Pix2Pix), the NWP (ground truth), and the PMs converted by the pre-trained and fine-tuned models. The fine-tuned model achieved the lowest RMSE across all tide gauges, with its maximum of only 0.12 m. In contrast, the PM and the pre-trained model reached the maximum RMSE of 0.38 m and 0.20 m, respectively.
- A storm surge hindcast for the extreme TC Faxai revealed that the fine-tuned model achieved the lowest RMSE at all 6 tide gauges, with its maximum of 0.32 m at Tokyo. Conversely, the PM exhibited the RMSE exceeding 0.5 m at Tokyo, where topographic effects on the wind field are predominant. The pre-trained model also failed to adequately capture the peak surges, resulting in a significant underestimation with the RMSE of up to 0.5 m in the inner bay (Chiba).

## Supplementary Information

Below is the link to the electronic supplementary material.

Supplementary Material 1

Supplementary Material 2

## Data Availability

The data supporting this study’s findings are available from the corresponding author upon reasonable request.
