# Supplementary material for: Physics-based data augmentation to enhance deep learning performance in tropical cyclone and storm surge modelling
Source: Sci Rep. 2026 Jun 22;16:28558. doi: 10.1038/s41598-026-58153-2 (PMC13572487; doi:10.1038/s41598-026-58153-2)
Supplement: Supplementary file 1 [file 41598_2026_58153_MOESM2_ESM.docx]

Supplementary Materials for

**Physics-based data augmentation to enhance deep learning performance in tropical cyclone and storm surge simulations**

Takumu Iwamoto^*1^ and Tomohiro Takagawa^1^

^1^ Tsunami and Storm Surge Group, Port and Airport Research Institute, Yokosuka, Japan.

* Correspondence to: [iwamoto-t@p.mpat.go.jp](mailto:iwamoto-t@p.mpat.go.jp)

This file includes:

Supplementary Text 1 to 4

Tables S1 to S2 (Table S3 provided as a separate CSV)

Figures S1 to S6

Description of Supplementary Data 1

References for Supplementary Information

**Introduction**

This document provides the results of the spin-up simulations conducted to generate initial conditions for the TC advection simulations, alongside further details on the validation of the fine-tuning process. Specifically, we describe the investigation into the optimal number of frozen layers and the training epochs required to achieve peak conversion performance during fine-tuning. Furthermore, to verify the accuracy of the simulated meteorological fields for TC Faxai (2019), we present comparisons between the observed and simulated time series of sea-level pressure and 10 m wind speed at various locations around Tokyo Bay.

**Supplementary Text** **1**: Spin-up Simulation Results

Following the method proposed by [1], spin-up simulations of the axisymmetric tropical cyclone (TC) model (RH87) [2] were performed. These simulations were conducted using a total of 81 parameter combinations, as detailed in Table S2. The resulting steady-state characteristics of the TCs are compiled in Table S3 (provided as a separate CSV file; see Supplementary Data 1). Fig. S1 shows scatter plots of the wind radii $r_{50}$ and $r_{30}$ against the central pressure $p_{c}$, based on these results. Among the 81 cases, a representative case of an extreme TC was selected for the subsequent advection simulations, characterized by a radius of maximum wind $r_{m}$ = 25 km, outer radius $r_{o}$ = 200 km, maximum wind speed $v_{m}$ = 45 m/s, and sea surface temperature $SST$ = 28 °C.

**Supplementary Text 2** : Selection of Blocks for Weight Updates in Fine-tuning

In the fine-tuning process, the changes in the generator loss function depending on the number of blocks subject to weight updates were examined for both training and validation data. For both datasets, when the number of updated blocks was small, a trend was observed where the loss function increased as training progressed (Fig. S2). It was found that when including all Encoder blocks up to the bottleneck block as targets for weight updates (E1-2-3-4-5-6-7-B), the loss function decreased stably. Updating the weights of all blocks showed a similar trend, and the loss function values after 200 epochs were comparable; however, the training time increased by a factor of approximately 1.3. Based on these results, the trained model obtained by updating all encoder blocks and the bottleneck block was used for subsequent investigations.

**Supplementary Text 3** : Determining the Optimal Training Epoch

As the performance of converting a parametric TC model (PM) using Pix2Pix depends heavily on the number of fine-tuning epochs, two criteria were adopted to determine the epoch showing the highest performance using the augmented validation data: the Skill Score [3] and the Artifact Index. These criteria are defined as follows:

$$\begin{aligned} Skill Score=1-\frac{\mathrm{MSE}\left( V^{Pix2Pix}, V^{\mathrm{Target}} \right)}{\mathrm{MSE}\left( V^{\mathrm{PM}}, V^{\mathrm{Target}} \right)}\#\left( S1 \right) \end{aligned}$$

$$\begin{aligned} Artifact Index=\mathrm{MAE}\left[ W_{i,j}^{Pix2Pix}-\mathrm{MED}_{3}\left( W_{i,j}^{Pix2Pix} \right) \right]\#\left( S2 \right) \end{aligned}$$

Here, $V$ is the simulated fields (i.e., sea level pressure, wind speed, or wind direction), $W$ is the wind speed, and the subscripts $i$ and $j$ denote the pixel coordinates. $\mathrm{MSE}$ is the mean squared error, $\mathrm{MAE}$ is the maximum absolute error, and $\mathrm{MED}_{3}$ is a median filter with a $3\times3$ kernel size. The Skill Score ranges from -1 to 1; a value closer to 1 indicates higher performance to Pix2Pix ($V^{Pix2Pix}$) relative to the baseline ($V^{\mathrm{PM}}$), with a score of 1 representing a perfect match to the ground truth $V^{\mathrm{Target}}$. The Artifact Index indicates the degree of deviation of a specific pixel from its neighbors; a value closer to 0 suggests that the wind fields converted by Pix2Pix are spatially smooth and free of noise.

Fig. S3 depicts the Skill Scores and the Artifact Index for the fine-tuned models at 10-epoch intervals. The results indicate that the 100-epoch case provides the best performance during both the entire simulation period and the TC passing phase (defined as the 6-hour window centered on the time of minimum sea level pressure). All simulated fields exhibited significant improvement relative to the PM ($Skill Score>0.3$), and the Artifact Index was the third lowest among all epochs. Based on these findings, we selected the model fine-tuned for 100 epochs as the optimal case.

**Supplementary Text 4** : TC track discrepancies

As shown in Fig. S4, the track discrepancies between the models (w/o DA and w/ DA) and the target (i.e., the PM) are of the order of 10 km for the reanalysis data. In the augmented dataset, these differences remain generally less than 20 km. These results indicate that while the reconstructed TC tracks deviate slightly from the PM—particularly for TCs with larger spatial scales (see Fig. 8)—the differences are negligible for compact TCs. Notably, there is no significant difference between the probability density functions of the w/o DA and w/ DA models. This suggests that the track discrepancies stem from the inherent characteristics of the Pix2Pix architecture rather than the effects of data augmentation. Specifically, since Pix2Pix operates on an image-to-image basis, discretized pixel-wise errors are inevitable. Given that the horizontal resolution in this study is 5 km per pixel, a slight shift of just a few pixels results in the observed track differences.

**Table S1**. Details of observation data used in this study.

| **Name** | **Type** | **Latitude** | **Longitude** | **Sampling interval** |
| --- | --- | --- | --- | --- |
| Tokyo | Tide | 35.650000N | 139.766667E | 15 Sec. |
| Chiba | Tide | 35.566667N | 140.050000E | 30 Sec. |
| Yokohama | Tide | 35.450000N | 139.650000E | 30 Sec. |
| Yokosuka | Tide | 35.283333N | 139.650000E | 30 Sec. |
| Kurihama | Tide | 35.227500N | 139.721111E | 6 Sec. |
| Dainikaiho | Tide | 35.316667N | 139.733333E | 1 Sec. |
| Tokyo | SLP | 35.691667N | 139.750000E | 10 min. |
| Chiba | SLP | 35.601667N | 140.103333E | 10 min. |
| Yokohama | SLP | 35.438333N | 139.651667E | 10 min. |
| Yokosuka | SLP | 35.281324N | 139.672085E | 10 min. |
| Tateyama | SLP | 34.986667N | 139.865000E | 10 min. |
| Kannonzaki | SLP | 35.255000N | 139.746667E | 10 min. |
| Umihotaru | Wind | 35.466167N | 139.871667E | 10 min. |
| Daini Kaiho | Wind | 35.312500N | 139.743889E | 10 min. |
| Uraga | Wind | 35.211667N | 139.786333E | 10 min. |

**Table S2**. Parameters for the RE87 model.

| **Parameter** | **Value** |
| --- | --- |
| Radius of maximum wind speed $r_{m}$ (km) | 25, 75, 125 |
| Outer radius of tropical cyclone $r_{o}$ (km) | 200, 500, 1000 |
| Maximum wind speed $v_{m}$ (m/s) | 35, 45, 55 |
| Sea surface temperature $SST$ (deg.) | 26, 28, 30 |

**Figure S1**. Relationship between central pressure and radius of 50 kt / 30 kt winds ($r_{50}$ / $r_{30}$), including results from the spin-up simulations. The definitions of "Reanalysis" and "Best Track" are the same as in Fig. 1. The same criteria as those for "Reanalysis" and "Best Track" were applied to extract these statistics. "Selected" indicates the case used in the subsequent advection simulations.

**Figure S2**. Changes in the generator loss function according to the number of blocks subject to weight updates. The blocks targeted for update and the corresponding training time are indicated in the upper-left labels.

**Figure S3**. Skill Scores and Artifact Index for the fine-tuned models at 10-epoch intervals using the augmented validation data. Blue and orange bars indicate the scores calculated for the entire simulation period and the TC approaching phase, respectively.

**Figure S4.** Probability density functions of the track difference from the target image for the test data in both w/o DA and w/ DA models.

**Figure S5**. Comparison of time series for sea level pressure. Results are shown for the PM used as the Pix2Pix input (Input), the pre-trained model (w/o DA), and the fine-tuned model (w/ DA). "Obs." indicates observed values. The lines and hatched areas indicate results obtained from the grid cells nearest to the weather stations and their adjacent grids.

**Figure S6**. Comparison of time series for 10 m wind speed. Results are shown for the PM used as the Pix2Pix input (Input), the pre-trained model (w/o DA), and the fine-tuned model (w/ DA). "Obs." indicates observed values. The lines and hatched areas indicate results obtained from the grid cells nearest to the weather stations and their adjacent grids.

**Supplementary Data 1**

The CSV file provides the comprehensive results of the one-week spin-up simulations for the 81 cases discussed in Supplementary Text 1. The first column lists the specific combinations of RE87 input parameters for each case. Subsequent columns summarize the simulated steady-state characteristics: maximum wind speed $v_{m}$, central pressure $p_{c}$, radius of 50 kt winds $r_{50}$, and radius of 30 kt winds $r_{30}$. $r_{50}$ is left blank if the wind field did not satisfy the threshold for extracting the wind radius.

**References**

[1] Rotunno, R. & Emanuel, K. A. An air–sea interaction theory for tropical cyclones. Part II: evolutionary study using a nonhydrostatic axisymmetric numerical model. *J. Atmos. Sci.* **44**, 542–561; 10.1175/1520-0469(1987)044<0542:AAITFT>2.0.CO;2 (1987).
[2] Bruyère, C. L. et al. Physically-based landfalling tropical cyclone scenarios in support of risk assessment. *Weather Clim. Extrem.* **26**, 100229; 10.1016/j.wace.2019.100229 (2019).
[3] Murphy, A. H. Skill Scores Based on the Mean Square Error and Their Relationships to the Correlation Coefficient. *Mon. Weather Rev.* **116**, 2417–2424; 10.1175/1520-0493(1988)116<2417:SSBOTM>2.0.CO;2 (1988)
